# Clinical implications of skincare: lessons from placebo-controlled dermatology trials

**DOI:** 10.1093/skinhd/vzag020

**Published:** 2026-04-02

**Authors:** Chawalpat Siripanich, Yan Ching Chow, Faisal R Ali

**Affiliations:** School of Medicine and Dentistry, University of Lancashire, Preston, UK; School of Medicine and Dentistry, University of Lancashire, Preston, UK; School of Medicine and Dentistry, University of Lancashire, Preston, UK; East Cheshire Dermatology Department, Mid Cheshire NHS Foundation Trust, Macclesfield, UK; Dermatological Surgery and Laser Unit, St John’s Institute of Dermatology, Guy’s and St Thomas’ NHS Foundation Trust, London, UK

## Abstract

Over the past decade, nonprescription skincare has gained significant attention. However, evidence supporting many skincare trends often lacks the rigorous testing seen in randomized controlled trials (RCTs) for prescribed medications. Vehicle arms in RCTs, which test nonmedicated treatments, provide insights that support the advocacy of basic skincare practices. This narrative literature review explores the lessons drawn from vehicle arms of RCTs in the management of conditions like acne vulgaris, actinic keratoses, melasma, postinflammatory hyperpigmentation and rosacea. A literature search was performed on MEDLINE and Embase to identify RCTs involving vehicle arms for the dermatological conditions. Data on outcomes, study design and sample size were extracted and analysed. Basic skincare practices such as cleansing, moisturizing and sun protection have consistently demonstrated meaningful improvements in dermatological outcomes, even without active treatments. Vehicle arms often achieve a substantial proportion of the efficacy seen in active treatments, frequently exceeding half of the efficacy seen in active treatments, emphasizing the potential of simple skincare regimens. For acne vulgaris, two identical international phase III RCTs (involving 2817 participants) demonstrated up to 25.7% improvement in facial acne severity and 51.2% reduction in inflammatory lesions with vehicle treatment. In actinic keratoses, an RCT (*n* = 468) showed up to 24% reduction in lesion count and 17% complete clearance in the vehicle arm. In melasma, an RCT (*n* = 48) demonstrated up to 66.6% improvement in severity with vehicle treatment. In postacne vulgaris postinflammatory hyperpigmentation, a phase IV trial (*n* = 123) showed up to 44.9% improvement in overall disease severity with vehicle treatment. For rosacea, a multicentre RCT (*n* = 120) showed up to 27% reduction in erythema in the vehicle arm. Basic skincare is an essential component of dermatological treatment. Evidence from placebo-controlled trials reinforces the value of skincare routines, which should be incorporated into treatment plans alongside active therapies to enhance efficacy and optimize patient outcomes.

## Teaser

Placebo-controlled dermatology trials reveal that vehicle arms (nonmedicated components) often yield clinically meaningful improvements in skin conditions like acne, melasma and rosacea. This review highlights how basic skincare practices, including cleansing, moisturizing and photoprotection, contribute significantly to treatment outcomes. These findings advocate for the integration of foundational skincare into standard dermatological care.

Over the past decade, there has been an inexorable increase in interest in nonprescription skincare across many sectors of society. However, the evidence underlying many of the latest skincare trends is often scant, and these proposed routines are typically not subjected to the same degree of rigour as the randomized controlled trials (RCTs) that are applied to prescribed medications. RCTs frequently include a vehicle arm to compare the effects of the active treatment against a ‘control group’ which receives the nonmedicated vehicle agent alone. The vehicle arm often provides insights into treatment of various dermatological conditions, which supports advocacy of basic skincare practices for these conditions.^1^

This literature review explores lessons drawn from the non­active arm of RCTs, highlighting their potential to support ­skincare practices in the management of conditions such as acne vulgaris, actinic keratosis (AK), hyperpigmentation and rosacea.

## Materials and methods

A literature search was performed on MEDLINE and Embase from inception to 31 December 2024 using the keywords ‘randomised controlled trial’ and several dermatological conditions, including ‘acne vulgaris’, ‘actinic keratoses’, ‘melasma’, ‘post-inflammatory hyperpigmentation’ and ‘rosacea’. The search focused on the outcomes measured in the vehicle arm of the studies. Studies were included if they were RCTs reporting outcomes for a vehicle arm, conducted in human participants and available as full-text articles in English. Studies were excluded if they were nonrandomized, case series or reviews; if they did not report outcomes for the vehicle arm; or if they were published only as conference abstracts, editorials or grey literature. Additionally, data on study design, sample size and treatment specifics were extracted.

## Definitions

### Vehicle

The base or carrier formulation in which the active ingredient is delivered. Vehicles often contain excipients such as emollients or humectants and may have intrinsic moisturizing, barrier-restoring or soothing effects independent of the active treatment.

### Relative vehicle effect

The proportion of clinical improvement in the vehicle arm compared with the active treatment arm, expressed as a percentage. This metric helps quantify the contribution of the vehicle alone. However, relative vehicle effect (RVE) values should be interpreted with caution, as they may be influenced by trial design, outcome measures, patient adherence and other contextual effects.

### Hawthorne effect

A behavioural change that occurs when participants modify their actions simply because they are aware they are being observed in a study. In dermatology trials, this may manifest as increased adherence to skincare routines or more careful self-care, independent of the intervention itself.

### Placebo effect

A perceived or actual improvement in symptoms arising from the expectation of benefit rather than the active properties of the treatment. In vehicle arms, this can contribute to clinical improvement even when no pharmacologically active ingredient is present.

### Expectancy effect

A form of bias where participants’ or investigators’ expectations about the outcome influence their perception or reporting of results. For example, clinicians grading lesion severity may unconsciously rate outcomes more favourably if they anticipate improvement.

## Results

### Acne vulgaris

Acne vulgaris, also known as acne, is a common dermatosis affecting the pilosebaceous unit, where clogged hair follicles become inflamed due to excess oil, dead skin cells and bacteria.^2^ The National Institute for Health and Care Excellence (NICE) has recommended incorporating skincare guidance, including cleansers, moisturizers and sunscreens, as part of the management plan for patients with acne.

In the PERFECT 1 and PERFECT 2 RCTs, international phase III studies (involving a total of 2817 participants) assessing the efficacy of the fourth-generation retinoid trifarotene 0.005% cream on facial and truncal acne, significant reductions in the severity of acne were demonstrated in patients randomized to the treatment arm of the study.^3^ However, notable improvements were also observed in the vehicle arm of the trial. Specifically, improvements were seen in patients in the vehicle arm, which involved cleansing the skin followed by applying the nonmedicated cream. In facial acne, the success rates [as gauged by the Investigator Global Assessment (IGA)] for the vehicle cream in PERFECT 1 and PERFECT 2 were 19.5% and 25.7% (compared with 29.4% and 42.3% in the active arm), and the mean percentage reductions from baseline of inflammatory lesions were 44.8% and 51.2% (compared with 54.4% and 66.2% in the active arm). For truncal acne, the success rates (as gauged by Physician Global Assessment) for the vehicle cream in PERFECT 1 and PERFECT 2 were 25.0% and 29.9% (compared with 35.7% and 42.6% in the active arm), and the mean percentage reductions from baseline of inflammatory lesions were 50.0% and 51.1% (compared with 57.4% and 65.4% in the active arm).

In another phase IV RCT assessing the efficacy of trifarotene 0.005% cream in facial acne, all patients (*n* = 123) were instructed to use a skin cleanser, moisturizer and sun protection factor (SPF) 30 sunscreen.^4^ The vehicle cream was applied nightly in the control arm. The improvement rate (as gauged by IGA) for the vehicle cream was 39.4% (compared with 61.1% in the active arm), and the mean percentage reduction from baseline of total acne lesions was 62.8% (compared with 72.0% in the active arm).

In a multicentre, split-face RCT assessing the efficacy of adapalene 0.3% and benzoyl peroxide 2.5% gel in facial acne, all patients (*n* = 67) were instructed to wash their faces with a foam wash in the morning and evening and apply SPF 30 moisturizer every morning.^5^ The vehicle gel was applied nightly in the control half. In this study, the vehicle arm showed an improvement rate (as gauged by IGA) of 19.4% (compared with 64.2% in the active arm), and the mean percentage reduction from baseline of inflammatory lesions was 57.9% (compared with 86.7% in the active arm).

In another multicentre, phase III study assessing the efficacy of retinaldehyde 0.1% and glycolic acid 6% in facial acne, all patients (*n* = 87) were instructed to cleanse the skin, avoid sun exposure and apply SPF 20 or SPF 60 sunscreen based on their Fitzpatrick skin phototype.^6^ The vehicle cream was applied nightly after cleansing in the control arm. The improvement rate (as gauged by Global Treatment Efficacy Scale) for the vehicle cream was 38.2% (compared with 55.3% in the active arm), and the mean percentage reduction from baseline of inflammatory lesions was 50.3% (compared with 53.8% in the active arm). It is crucial to note that the differences in inflammatory lesion reductions were statistically insignificant, indicating that the vehicle arm performed just as well as the active arm.

Additionally, findings from the START study, a phase IV, multicentre, split-face RCT, demonstrated that consistent use of a basic skincare routine (including a gentle cleanser, moisturizer and broad-spectrum SPF) significantly reduced the development of new atrophic acne scars in young individuals with active acne.^7^ These results further emphasize the long-term protective role of nonprescription skincare in acne management and its potential to prevent sequelae beyond inflammatory lesion burden.

### Actinic keratoses

AKs are precancerous skin lesions that develop due to prolonged sun exposure, typically appearing as rough, scaly patches on areas of the skin exposed to the sun.^8^ Two studies support the use of moisturizers for reduction of AKs. In a multicentre, parallel-group RCT involving 195 participants, the efficacy of diclofenac 3.0% in hyaluronic acid 2.5% gel was evaluated as a treatment for AK with a 1:1:1:1 randomization pattern.^9^ Participants were assigned to receive either the treatment for 30 days, the treatment for 60 days, the vehicle for 30 days or the vehicle for 60 days, applied twice daily. The mean percentage reductions from baseline in the number of target lesions for the vehicle arm in the 30-days group and 60-days group were both 33.8% (compared with 57.9% and 55.7% in the active arm).

Underlining the importance of sun protection, in a multicentre RCT assessing the efficacy of fluorouracil 5% cream in AK, all patients (*n* = 468) were instructed to use SPF 30 sunscreen in the morning and received education on sun protection.^10^ The vehicle cream was applied twice daily in the control arm. In the vehicle arm, the mean percentage reduction from baseline of AK count was 24% (compared with 73% in active arm), and the percentage of patients achieving complete clearance of AK was 17% (compared with 38% in the active arm).

### Hyperpigmentation

Melasma is a skin condition characterized by dark, discoloured patches, usually on the face, caused by an overproduction of melanin, often triggered by sun exposure and hormonal changes.^11^ Sunscreen protection is a mainstay of the management of patients with melasma.

In an RCT assessing the efficacy of isotretinoin 0.05% gel in melasma, all patients (*n* = 30) were instructed to apply SPF 28 sunscreen in the morning.^12^ The vehicle gel was applied daily in the control arm. In the vehicle arm, the mean percentage reductions from baseline were 60% using Melasma Area and Severity Index (MASI) and 34% using Melasma Area and Melanin Index (MAMI) (compared with 68.2% for MASI and 47% for MAMI in the active arm).

In another RCT assessing the efficacy of hydroquinone 4% cream in melasma, all patients (*n* = 48) were instructed to use a cleanser twice daily and a SPF 30 sunscreen in the morning.^13^ The formulations for both active and vehicle included SPF 15 sunscreen. The vehicle cream was applied twice daily in the control arm. In the vehicle arm, the percentage of participants achieving partial-to-total improvement (as gauged by clinical observations) was 66.6% (compared with 95.3% in the active arm).

Postinflammatory hyperpigmentation (PIH) is a condition where dark spots or patches develop on the skin after an inflammatory skin event due to an overproduction of melanin in response to the inflammation.^14^

In a phase IV RCT assessing the efficacy of trifarotene 0.005% cream in postacne vulgaris PIH, all patients (*n* = 123) were instructed to use a gentle skin cleanser, moisturizer and SPF 30 sunscreen.^4^ The vehicle cream was applied daily at night in the control arm. The mean percentage reduction from baseline of overall disease severity score was 44.9% (compared with 45.4% in the active arm), and the median percentage reduction from post–acne vulgaris hyperpigmentation index was 11.3% (compared with 18.9% in the active arm).

In another RCT assessing the efficacy of clindamycin phosphate 1.2% and tretinoin 0.025% gel, all patients (*n* = 60) were instructed to use skin cleanser twice daily and SPF 30 sunscreen in the morning.^15^ The vehicle cream was applied nightly in the control arm. The mean percentage reduction from baseline of PIH severity scale score was 26.2% (compared with 36.5% in the active arm).

### Rosacea

Rosacea is a chronic skin condition characterized by facial redness, visible blood vessels, and sometimes pimples or thickened skin.^16^ NICE has recommended the utility of gentle cleansers, moisturizers, sunscreens and sun avoidance in the management of patients with rosacea.

Two identical large-scale, international phase III studies, designated ‘study 1’ and ‘study 2’ (involving a total of 1371 participants), demonstrated the positive effects of ivermectin 1% cream in the treatment of rosacea.^17^ Patients in the control arm were only instructed to apply vehicle cream once nightly. The success rates (as gauged by IGA) for the vehicle cream in ‘study 1’ and ‘study 2’ were 11.6% and 18.8% (compared with 38.4% and 40.1% in the active arm); the median percentage reduction from baseline of inflammatory lesions was 50% in both studies (compared with 76.0% and 75.0% in the active arm); and participant satisfaction (as gauged by assessment questionnaire) was 38.6% and 34.4% (compared with 69.0% and 66.2% in the active arm).

In another multicentre RCT assessing metronidazole 1% cream in rosacea, patients (*n* = 120) were instructed to apply the treatment or the vehicle twice daily.^18^ Both the formulations contained SPF 15 sunscreen. The mean percentage reduction from baseline of erythema score was 27% (compared with 42% in the active arm), and the median percentage reduction from baseline of inflammatory lesions was 25.4% (compared with 65.1% in the active arm). A consolidated overview of trial outcomes and associated RVE values is presented in Table 1.

**Table 1:** Summary of outcomes and relative vehicle effect by condition

Condition	Study	No. of patients	Placebo arm	Active arm	Outcome measured	Vehicle effect (%)	Active effect (%)	RVE (%)
Acne vulgaris	Tan^3^	2817	Cleanser, vehicle cream	Cleanser, trifarotene 0.005% cream	IGA success rates	19.5	29.4	66.3
						25.7	42.3	60.8
					Mean percentage reduction from baseline of inflammatory lesions	44.8	54.4	82.4
						51.2	66.2	77.3
					PGA success rates (truncal)	25.0	35.7	70.0
						29.9	42.6	70.2
					Mean percentage reduction from baseline of inflammatory lesions (truncal)	50.0	57.4	87.1
						51.1	65.4	78.1
	Alexis^4^	123	Cleanser, moisturizer, SPF 30 sunscreen, vehicle cream	Cleanser, moisturizer, SPF 30 sunscreen, trifarotene 0.005% cream	IGA improvement rate	39.4	61.1	64.5
					Mean percentage reduction from baseline of total acne lesions	62.8	72.0	87.2
	Dréno^5^	67	Cleaser, SPF 30 moisturizer	Cleaser, SPF 30 moisturizer, adapalene 0.3% and benzoyl peroxide 2.5% gel	IGA improvement rate	19.4	64.2	30.2
					Mean percentage reduction from baseline of inflammatory lesions	57.9	86.7	66.8
	Poli^6^	87	Cleanser, sun avoidance, SPF 20 or 60 sunscreeen based on phototype	Cleanser, sun avoidance, SPF 20 or 60 sunscreeen based on phototype, retinaldehyde 0.1% and glycolic acid 6%	GTES improvement rate	38.2	55.3	69.1
					Mean percentage reduction from baseline of inflammatory lesions	50.3	53.8	93.5
Actinic keratoses	Rivers^9^	195	Vehicle gel	Diclofenac 3.0% in hyaluronic acid 2.5% gel	Mean percentage reduction from baseline in the number of target lesions (30 days arms)	33.8	57.9	58.4
					Mean percentage reduction from baseline in the number of target lesions (60 days arms)	33.8	55.7	60.7
	Pomerantz^10^	468	SPF 30 sunscreen, education on sun protection, vehicle cream	SPF 30 sunscreen, education on sun protection, fluorouracil 5% cream	Mean percentage reduction from baseline of AK count	24.0	73.0	32.9
					Percentage of patients achieving complete clearance of AK	17.0	38.0	44.7
Melasma	Leenutaphong V.^12^	30	SPF 28 sunscreen, vehicle gel	SPF 28 sunscreen, isotretinoin 0.05% gel	Mean percentage reductions from baseline (MASI)	60.0	68.2	88.0
					Mean percentage reductions from baseline (MAMI)	34.0	47.0	72.3
	Ennes^13^	48	Cleanser, SPF 30 sunscreen, vehicle cream (SPF 15)	Cleanser, SPF 30 sunscreen, hydroquinone 4% cream (SPF 15)	Percentage of participants achieving partial-to-total improvement (clinical observation)	66.6	95.3	69.9
PIH	Alexis^4^	123	Cleanser, moisturizer, SPF 30 sunscreen, vehicle cream	Cleanser, moisturizer, SPF 30 sunscreen, trifarotene 0.005% cream	Mean percentage reduction from baseline of overall disease severity score	44.9	45.4	98.9
					Median percentage reduction from post-acne vulgaris hyperpigmentation index	11.3	18.9	59.8
	Callender^15^	60	Cleanser, SPF 30 sunscreen, vehicle cream	Cleanser, SPF 30 sunscreen, clindamycin phosphate 1.2% and tretinoin 0.025% gel	Mean percentage reduction from baseline of PIH severity scale score	26.2	36.5	71.8
Rosacea	Stein^17^	1371	Vehicle cream	Ivermectin 1% cream	IGA success rates	11.6	38.4	30.2
						18.8	40.1	46.9
					Median percentage reduction from baseline of inflammatory lesions	50.0	76.0	65.8
						50.0	75.0	66.7
					Participant satisfaction	38.6	69.0	55.9
						34.4	66.2	52.0
	Nielsen^18^	120	Vehicle cream (SPF 15)	Metronidazole 1% cream (SPF 15)	Mean percentage reduction from baseline of erythema score	27.0	42.0	64.3
					Median percentage reduction from baseline of inflammatory lesions	25.4	65.1	39.0

AK, actinic keratosis; GTES, Global Treatment Efficacy Scale; IGA Investigator Global Assessment; MAMI, Melasma Area and Melanin Index; MASI, Melasma Area and Severity Index; PGA, Physician Global Assessment; PIH, postinflammatory hyperpigmentation; RVE, relative vehicle effect; SPF, sun protection factor.

## Discussion

For all conditions assessed, basic skincare practices (cleansing, moisturizing and using sunscreen) with nonmedicated vehicles consistently led to meaningful improvements, underscoring the therapeutic value of foundational skincare. However, it is important to note that the exact composition of skincare regimens varied across studies, with some incorporating only one or two components.

Placebo-controlled trials consistently demonstrate that good, basic skincare practices alone contribute significantly to clinical improvement in dermatological outcomes, emphasizing the intrinsic value of foundational skincare practices. Evidence from trials in which only one or two elements of the foundational skincare triad are employed demonstrates that even a partial regimen can still elicit clinically meaningful therapeutic benefit. While active treatments surpass vehicle controls, the latter achieve at least half of the efficacy observed in active arms in many cases, as outlined in Table 1. This heavily underlines the therapeutic potential of simple skincare routines and their roles as adjuvants to increase efficacy in treatment. However, RVE values should be interpreted with caution, as they may be influenced by trial design, outcome measures, patient adherence and other contextual effects. Patients may often overlook the importance of basic skincare practices, as these are not medications. A survey of patients with rosacea (*n* = 2340) found that sun exposure and temperature changes (60% and 69%, respectively) were the most frequent triggers for worsening their rosacea.^19^ Despite this, only 5% included sunscreen in their skincare routine.

Although therapeutic interventions are central to the management of inflammatory dermatoses, adjunctive use of appropriate cleansing, moisturization and photoprotection plays an equally important role in optimizing clinical outcomes.^20^ Cleansing is the initial step to eliminate dirt, pollutants, oil and sweat, dead skin cells and bacteria, which can exacerbate skin issues if not adequately addressed.^21,22^ Moisturizers hydrate the skin and help lock in moisture, while also contributing to skin barrier repair, pH regulation and microbiome balance. Specifically, ceramide-containing formulations have been shown to normalize skin pH and reduce inflammation, in addition to the barrier-supportive benefits. This is particularly important in conditions like acne, where both barrier dysfunction and inflammation play key roles in disease pathogenesis. Sunscreens protect the skin from harmful ultraviolet radiation, which can cause inflammation and premature ageing, and increase the risk of skin cancer. These steps are generally well-tolerated, with minimal adverse effects, particularly in comparison with active treatment ingredients. Beyond improvement in inflammatory lesions and severity scores, basic skincare may also prevent long-term sequelae. This underscores the importance of early intervention with foundational skincare not only in active management, but also in long-term complication prevention. As such, all patients should begin with this basic regimen, as it can lead to meaningful improvements in skin health.

Dermatologists play a key role in incorporating these practices into everyday care by ensuring that patients understand the benefits of consistent and proper skincare, which can profoundly complement and enhance the results of specific dermatological treatments. This includes recommending suitable, gentle skincare products and educating patients on their correct usage. Patient education is essential in ensuring adherence to these fundamental steps, emphasizing the importance of consistency for optimal outcomes. However, it should be made clear that while a good skincare routine is an important part of the management plan, active treatment is still necessary, and patients should not rely solely on these basic practices or attempt self-medication. while these findings suggest potential benefits beyond facial disease, data for truncal and other body sites remain limited, warranting further research.

It is also important to consider that vehicle arms may show efficacy due to factors such as intrinsic properties of the vehicle, the skincare regimen itself and psychological influences such as placebo, Hawthorne and expectancy effects, or a combination of all of these. The magnitude of these effects likely varies across trials depending on design features such as blinding, use of intraindividual controls and objective outcome measures. While some studies attempted to reduce bias through blinded assessment or photographic evaluation, most relied heavily on subjective clinical grading, leaving results susceptible to expectancy effects and investigator bias. This may lead to inflated efficacy estimates in both active and vehicle arms due to subconscious expectation or grading variability. This heterogeneity limits direct comparability across studies and complicates interpretation of vehicle efficacy. Interpretation of vehicle arm outcomes must be approached with caution due to variability in vehicle composition, study design and outcome assessment methods. A true no-intervention arm would be required to isolate the specific contribution of the vehicle itself.

Adherence to skincare regimens in RCTs is closely monitored, often through diaries, reminders or investigator oversight, which may not reflect real-world behaviour. Consequently, the improvements attributed to vehicle regimens in trials may represent an upper bound of their effectiveness in everyday patient populations. Nonetheless, patients who remain diligent and consistent with basic skincare practices are likely to experience meaningful benefits, even if real-world outcomes may not fully mirror those observed under controlled conditions. This factor highlights the role of patient responsibility in optimizing outcomes.

## Conclusion

Skincare should be regarded as an essential and fundamental part of dermatological treatment. The evidence from placebo-controlled trials reinforces the significant clinical impact of basic skincare routines, supporting their value even in the absence of active, prescribed treatment. Dermatologists should actively incorporate guidance on skincare as a core part of treatment plans, advising patients to use appropriate cleansing, moisturization and photoprotection alongside active therapies. This approach not only enhances the efficacy of active treatments and improves overall skin health, but also helps to best emulate the results seen in clinical trials, ensuring optimal patient outcomes. Basic skincare should be viewed not merely as an adjunct, but as a low-risk, evidence-based therapeutic foundation in the management of common inflammatory dermatoses and prevention of their long-term sequelae.
